# Are template prostate biopsies still required in patients with high‐risk PI‐RADS lesions? A retrospective study of 470 patients

**DOI:** 10.1002/bco2.70286

**Published:** 2026-10-06

**Authors:** Josh Tiro, Christian Kirk, Finley Breeze, Amir Zarrabi, Luiz Gustavo Modelli de Andrade, Paulo Roberto Kawano, Flavio Vasconcelos Ordones, Lodewikus Vermeulen

**Affiliations:** ^1^ Department of Urology Tauranga Public Hospital Tauranga New Zealand; ^2^ Nephrology Division São Paulo State University (UNESP) Botucatu São Paulo Brazil; ^3^ Urology Division São Paulo State University (UNESP) Botucatu São Paulo Brazil; ^4^ Faculty of Health and Medical Sciences University of Auckland Auckland New Zealand

**Keywords:** image‐guided biopsy, magnetic resonance imaging, PI‐RADS, prostate cancer, prostate‐specific antigen density, systematic biopsy, targeted biopsy, transperineal biopsy

## Abstract

**Objectives:**

To determine the incremental diagnostic yield of systematic template sampling during transperineal prostate biopsy (TPPB) in biopsy‐naïve men and to identify patient subgroups where systematic biopsy might be safely omitted.

**Patients and Methods:**

We retrospectively analysed 470 consecutive biopsy‐naïve men with PI‐RADS 3–5 lesions on multiparametric MRI who underwent combined MRI‐targeted and systematic template TPPB between January 2022 and May 2026 at a single institution. Clinically significant prostate cancer (csPCa) was defined as ISUP grade group 2 or higher. The primary outcome was csPCa detected on template biopsy in men with non‐significant disease (ISUP < 2) on targeted biopsy.

**Results:**

Overall, csPCa was detected in 356/470 men (75.7%). Systematic template biopsy identified csPCa missed by targeted biopsy in 10/470 men (2.1%, 95% CI 1.0%–3.9%), representing 2.8% of all csPCa diagnoses. Smaller index lesion size on MRI was the only statistically significant predictor of csPCa missed by targeted biopsy (OR 0.50 per 5 mm increase, 95% CI 0.28–0.90, *p* = 0.021). In subgroup analysis cross‐tabulating PI‐RADS category and PSA density (PSAD), template biopsy provided zero incremental yield in patients with PI‐RADS 5 lesions and a PSAD > 0.20 ng/mL^2^ (0.0%, one‐sided 95% upper bound 2.5%). The highest incremental yield was observed in men with PI‐RADS 4 lesions (3.7%, 95% CI 1.7%–7.0%).

**Conclusions:**

In this biopsy‐naïve cohort of men undergoing combined MRI‐targeted and template TPPB, systematic template biopsy provided a small incremental diagnostic yield for csPCa (2.1%) overall, with no added value in men with PI‐RADS 5 lesions and a PSAD > 0.20 ng/mL^2^. A risk stratified approach to systemic sampling in these men may be considered. Validation in larger, multicentre cohorts is warranted before these findings can be incorporated into future guideline recommendations.

## 1 INTRODUCTION

Diagnostic pathways for prostate cancer continue to evolve. Evolution is driven by advances in PSA‐derived biomarkers, genetic testing, validated risk calculators and multiparametric magnetic resonance imaging (mpMRI), which have enabled more selective patient referral for biopsy. Concurrently, both the biopsy approach (transrectal versus transperineal) and biopsy strategy (systematic versus MRI‐targeted sampling) are undergoing considerable scrutiny.^1^

The PRECISION trial found that MRI‐targeted biopsy of PI‐RADS (Prostate Imaging Reporting and Data System) lesions alone detects 12% *more* clinically significant prostate cancer (csPCa) than systematic biopsy alone in biopsy‐naïve men, while also detecting 13% *less* clinically insignificant cancer.^2^, ^3^ However, the PRECISION trial compared targeted biopsy with systematic biopsy as alternative strategies; it did not establish the additional value of systematic sampling once a targeted biopsy has already been performed. Given this, the role of systematic biopsy in the presence of a high PI‐RADS (4 or 5) lesion has been increasingly questioned. Evidence shows that when performed alongside targeted biopsies, systematic biopsies identify 2%–11% additional csPCa depending on population, biopsy route and prior biopsy status.

A large multicentre European cohort found that systematic biopsy increased csPCa detection by only 5.8% (with 42% of biopsies performed transrectally), falling to 1.5% when restricted to contralateral cores,^4^ whereas a predominantly transrectal UK series (68% transrectal), which included men with prior negative or low‐grade biopsies, reported csPCa missed by targeted biopsy in 9%–11% of cases.^5^

For PI‐RADS 5 lesions specifically, one cohort reported that systematic biopsy still added a further 6.9% of csPCa diagnoses overall, with PSA density (PSAD) above 0.17 ng/mL^2^ identified as a predictor of finding cSPCa.^6^ This heterogeneity makes it difficult for clinicians to know, in their own practice and patient population, whether and in whom systematic biopsy can reasonably be reduced or omitted.

Worldwide, most clinical guidelines from large urology associations still suggest we continue with both target and some form of additional biopsy. European Association of Urology (EAU) suggest combining targeted with perilesional sampling in men with a positive MRI, whereas the American Urological Association (AUA) recommend targeted biopsy ± systematic biopsy.^7^ Most published series have studied transrectal fusion biopsy; relatively few have examined this question in transperineal prostate biopsy (TPPB), which is now favoured in many centres because of its lower infectious risk and can now be performed in the outpatient setting.^8^ Furthermore, few series have combined PI‐RADS category with other variables (such as PSAD) to define specific low‐risk combinations in which systematic biopsy might be safely omitted.

We therefore retrospectively analysed a single‐centre cohort of men undergoing combined MRI‐targeted and systematic TPPB to determine the incremental diagnostic value of systematic biopsy.

## 2 PATIENTS AND METHODS

### 2.1 Study design, setting and participants

We performed a retrospective cohort study of consecutive men who underwent an index, combined targeted and systematic template TPPB at Tauranga Hospital, New Zealand, between January 2022 and May 2026. All men with at least one PI‐RADS 3–5 lesion on pre‐biopsy multiparametric MRI were included in the study. Data was extracted from a prospectively maintained institutional biopsy database. 801 TPPB were performed during the study period. A total of 149 men were excluded as they did not have a PI‐RADS 3–5 lesion on multiparametric MRI. A further 135 men were excluded due to having had prior prostate biopsies. Finally, 47 men were excluded due to having only targeted OR template biopsies performed. The final cohort comprised 470 men (Figure 1).

**FIGURE 1:**
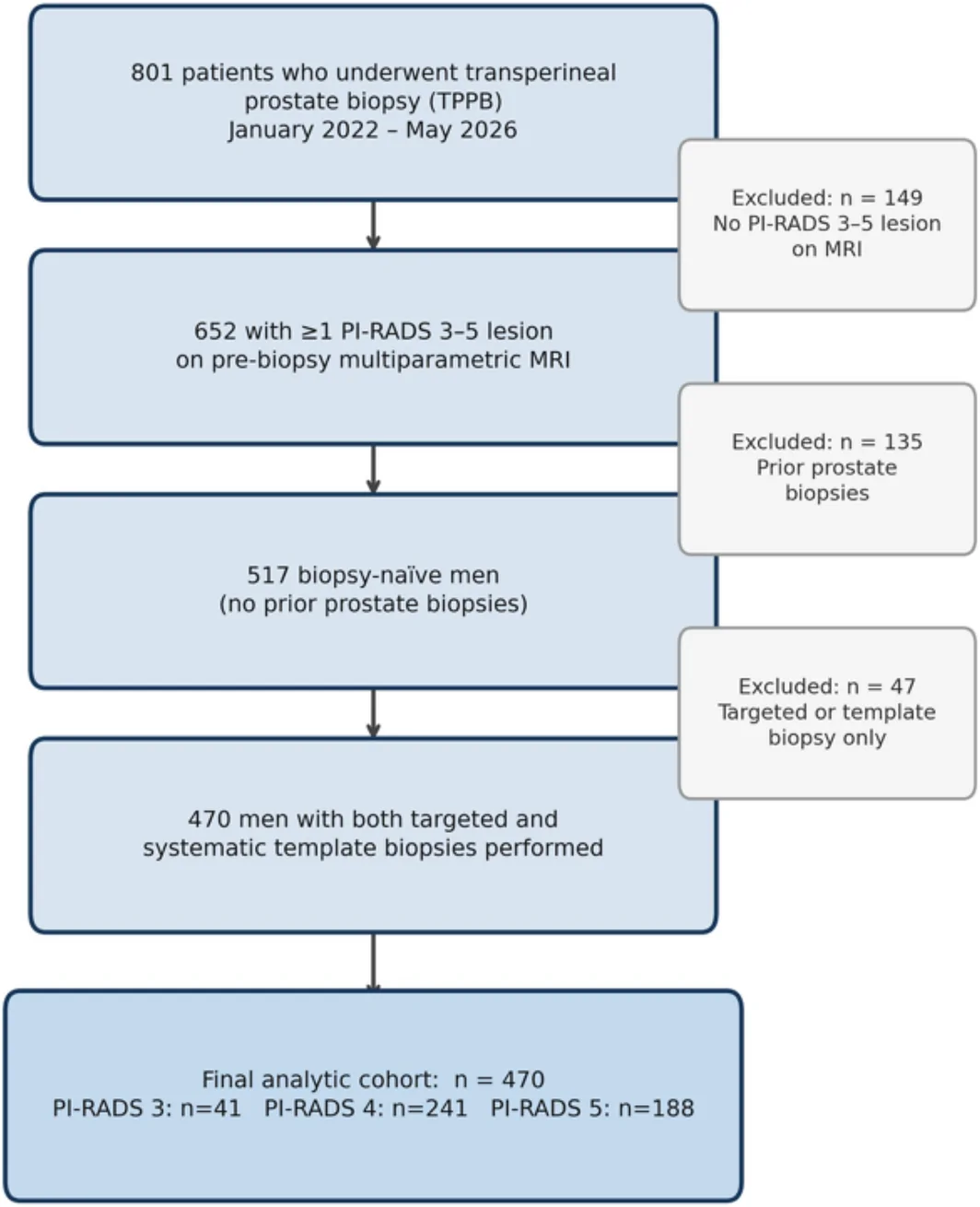
STROBE flowchart demonstrating exclusions to form final analytic cohort.

### 2.2 MRI acquisition and lesion characteristics

All men underwent pre‐biopsy multiparametric MRI reported according to PI‐RADS v2.1^9^; the highest‐scoring lesion was taken as the index lesion for each patient. Lesion size (maximum diameter, mm) and location (anterior, peripheral, transitional or central) were recorded from the MRI report. Prostate volume was derived from MRI, and PSAD was calculated as PSA divided by prostate volume.

### 2.3 Biopsy technique

Transperineal biopsy was performed in all cases, under local anaesthetic in an outpatient clinic and under general anaesthetic in theatre. Targeted cores were obtained from each PI‐RADS lesion using MRI‐guided cognitive fusion, with the aid of the PrecisionPoint system. Systematic cores were obtained as per the modified Ginsburg protocol.^10^

Targeted and template cores were processed and reported separately by biopsy location. For each patient, the highest ISUP grade group among targeted cores defined the targeted‐biopsy result, and the highest ISUP grade group among template cores defined the template‐biopsy result. csPCa was defined as ISUP grade group ≥ 2 (Gleason score ≥ 7 [3 + 4]).

### 2.4 Outcomes

**Primary outcome:** To identify the number of men with csPCa detected on **template biopsy** that had NO csPCa on **targeted biopsy** (i.e., template ISUP ≥ 2 and targeted ISUP < 2).

**Secondary outcomes:**

- any ISUP upgrade on template relative to targeted biopsy (template ISUP > targeted ISUP),
- the side (ipsilateral or contralateral to the index PI‐RADS lesion) of csPCa detected only on template biopsy
- to identify other independent predictors of the primary outcome, considering factors such as PI‐RADS grade, PI‐RADS lesion size and PSAD.

### 2.5 Statistical analysis

Continuous variables are summarised as median (interquartile range, IQR) and categorical variables as number (%). Proportions with csPCa (ISUP ≥ 2) detected only on template biopsy were reported with exact (Clopper–Pearson) 95% confidence intervals. For low‐risk subgroups with zero observed events, a one‐sided exact 95% upper confidence bound (the conservative maximum plausible miss rate) was calculated as the 95th percentile of a Beta (1, *n*) distribution. Stratified analyses were performed by PI‐RADS category, PSAD group (< 0.15, 0.15–0.20, > 0.20 ng/mL^2^) and index lesion size (< 10 mm, 10–20 mm, > 20 mm), with cross‐tabulations of PI‐RADS × PSAD and PI‐RADS × lesion size.

Given that only 10 primary‐outcome events were observed, no multivariable regression model of the primary outcome was undertaken, as this falls well below the conventional threshold of approximately 10 events per predictor and would risk an overfitted, unstable model. Associations between individual candidate predictors (index lesion size, prostate volume, PI‐RADS category, PSA, PSAD and age) and the primary outcome are instead reported as unadjusted (univariable) logistic regression estimates. An exploratory multivariable logistic regression model was additionally fitted for the secondary outcome of any ISUP upgrade on template biopsy (38 events), using PI‐RADS category, PSAD, age and prostate volume as covariates; this model should be regarded as hypothesis‐generating. Laterality (ipsilateral vs. contralateral) was assessed using the exact binomial test against an expected 50:50 distribution. All statistical analyses were performed in R Version 4.3.3 (R Foundation for Statistical Computing, Vienna, Austria) using the tidyverse, binom and gtsummary packages.

### 2.6 Ethical considerations

Ethical approval was obtained through the Local Research Approval authority, as well as through the Health and Disability Committee.

## 3 RESULTS

### 3.1 Patient characteristics

Of the 470 men in our cohort, the median age was 67 years (IQR 61–70) and median PSA was 7.8 ng/mL (IQR 5.2–12.0). Most men identified as European (77.4%) or New Zealand Māori (19.8%).

A PI‐RADS 4 lesion was the most common MRI finding (241/470, 51.3%), followed by PI‐RADS 5 (188/470, 40.0%) and PI‐RADS 3 (41/470, 8.7%).

Full baseline characteristics are shown in Table 1.

**TABLE 1:** Patient characteristics by PI‐RADS category.

Characteristic	PI‐RADS 3 (*n* = 41)	PI‐RADS 4 (*n* = 241)	PI‐RADS 5 (*n* = 188)	*p*‐value
Demographics				
Age, years, median (IQR)	62 (56–66)	66 (61–69)	68 (64–74)	< 0.0001*
BMI, kg/m^2^, median (IQR)	27.0 (25.8–30.0) *n* = 35	28.0 (25.8–31.0) *n* = 214	29.0 (26.1–32.3) *n* = 166	0.031*
Ethnicity—European	36 (87.8%)	194 (80.5%)	134 (71.3%)	
Ethnicity—Māori	3 (7.3%)	42 (17.4%)	48 (25.5%)	0.019†
Ethnicity—Asian	2 (4.9%)	3 (1.2%)	3 (1.6%)	
Ethnicity—Pasifika	0 (0%)	1 (0.4%)	2 (1.1%)	
Ethnicity—other	0 (0%)	1 (0.4%)	1 (0.5%)	
Laboratory and MRI characteristics				
PSA, ng/mL, median (IQR)	6.6 (4.5–8.3)	6.5 (4.9–10.0)	10.0 (6.8–17.0)	< 0.0001
Prostate volume, mL, median (IQR)	40 (32–60)	40 (30–50)	40 (32–54)	0.217
PSA density, ng/mL^2^, median (IQR)	0.138 (0.107–0.196)	0.182 (0.121–0.255)	0.253 (0.162–0.403)	< 0.0001
PSA density—< 0.15	24 (58.5%)	90 (37.3%)	40 (21.3%)	
PSA density—0.15–0.20	9 (22.0%)	49 (20.3%)	30 (16.0%)	< 0.0001
PSA density—> 0.20	8 (19.5%)	102 (42.3%)	118 (62.8%)	
Index lesion size, mm, median (IQR)	3.0 (3.0–3.0) *n* = 2	10.0 (8.0–12.0) *n* = 241	20.0 (17.5–25.5) *n* = 187	< 0.0001
Lesion location—peripheral	35 (85.4%)	184 (76.3%)	118 (62.8%)	
Lesion location—anterior	4 (9.8%)	51 (21.2%)	63 (33.5%)	< 0.0001
Lesion location—central	0 (0%)	2 (0.8%)	7 (3.7%)	
Lesion location—transitional	2 (4.9%)	3 (1.2%)	0 (0%)	
Lesion location—not recorded	0 (0%)	1 (0.4%)	0 (0%)	
Biopsy characteristics				
Targeted cores taken, *n*, median (IQR)	6 (5–7)	6 (5–8)	6 (5–9)	0.384
Template cores taken, *n*, median (IQR)	14 (9–16)	13 (10–17)	11 (8–15)	< 0.0001
Total cores taken, *n*, median (IQR)	19 (16–22)	19 (16–24)	18 (14–22)	0.001
Complications—none	38 (92.7%)	231 (95.9%)	183 (97.3%)	
Complications—vasovagal episode	3 (7.3%)	4 (1.7%)	2 (1.1%)	
Complications—infection	0 (0%)	2 (0.8%)	3 (1.6%)	
Complications—bleeding	0 (0%)	3 (1.2%)	0 (0%)	

*Note*: Data are median (interquartile range, IQR) for continuous variables, or *n* (column %) for categorical variables, by highest PI‐RADS category; denominators are 41 (PI‐RADS 3), 241 (PI‐RADS 4) and 188 (PI‐RADS 5) unless otherwise noted (BMI and index lesion size have additional missing data, concentrated in PI‐RADS 3 for lesion size).

### 3.2 Rate of any ISUP grade upgrade on template biopsy

Any ISUP grade upgrade on template biopsy relative to targeted biopsy (template ISUP > targeted ISUP) occurred in **38/470 men (8.1%, 95% CI 5.9%–10.9%)**.

Of these 38 upgrades, only 10 (26.3% of upgrades; 2.1% of the whole cohort) crossed the csPCa threshold and constitute the primary outcome above. A further 20 (52.6% of upgrades; 4.3% of the cohort) already had csPCa on targeted biopsy and template biopsy simply revealed an even higher ISUP grade, which may still influence risk stratification, peri‐operative staging considerations and treatment planning. The remaining 8 upgrades (21.1% of upgrades; 1.7% of the cohort) progressed from a negative targeted biopsy to ISUP grade group 1 on template. The overall composition of csPCa detection by biopsy method across the whole cohort is shown in Figure 2.

**FIGURE 2:**
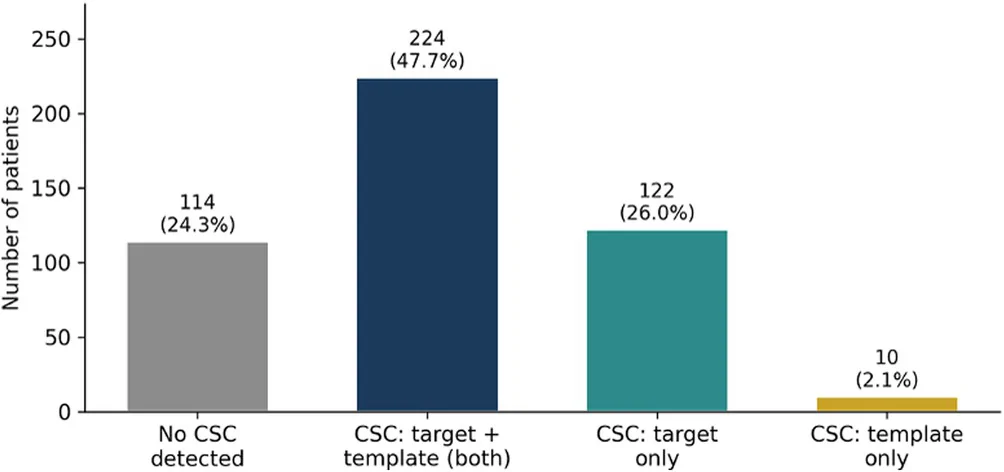
Composition of clinically significant cancer (ISUP grade group ≥ 2) detection across the cohort (*N* = 470), by biopsy method. ‘Template only’ corresponds to the primary outcome.

Overall, targeted biopsy showed a higher ISUP grade than template biopsy in 187/470 men (39.8%), template biopsy was higher in 38/470 men (8.1%) and the ISUP score was the same in 245/470 men (52.1%).

### 3.3 Ipsilateral versus contralateral distribution of template‐exclusive cancer

Among the 10 men with csPCa detected only on template biopsy, the additional cancer was located ipsilateral to the index PI‐RADS lesion in 5 (50%) and contralateral in 5 (50%; *p* = 1.00, 95% CI = 23.7%–76.3%). This should be interpreted cautiously given the small number of events.

In the larger group of 38 men with any ISUP upgrade on template biopsy, the additional or higher‐grade disease was ipsilateral in 28 (73.7%) and contralateral in only 10 (26.3%; *p* = 0.005), suggesting that ipsilateral template biopsies are more likely to detect prostate cancer, although a meaningful minority (26.3%) are contralateral.

### 3.4 Incremental value across PI‐RADS category and PSAD group

Our rates of csPCa detection with a combined biopsy strategy were 26.8%, 71.4% and 92% for PI‐RADS category 3, 4 and 5, respectively (Table 2).

**TABLE 2:** Detection rate of clinically significant prostate cancer (csPCa, ISUP grade group ≥ 2) by PI‐RADS category, shown separately for targeted biopsy alone, template (systematic) biopsy alone and combined (target + template) biopsy. 95% confidence intervals for the combined‐biopsy rate: PI‐RADS 3, 15.7%–41.9%; PI‐RADS 4, 65.4%–76.7%; PI‐RADS 5, 87.3%–95.1%.

PI‐RADS	*n*	csPCa on target biopsy	csPCa on template only	Combined biopsy (any csPCa)
3	41	11/41 (26.8%)	0/41 (0%)	11/41 (26.8%)
4	241	163/241 (67.6%)	9/241 (3.7%)	172/241 (71.4%)
5	188	172/188 (91.5%)	1/188 (0.5%)	173/188 (92.0%)
**Overall**	**470**	**346/470 (73.6%)**	**10/470 (2.1%)**	**356/470 (75.7%)**

Subgroup analyses were performed to identify the impact of PI‐RADS category and PSAD on the performance of template biopsies.

The primary outcome rate varied by PI‐RADS category: 0/41 (0%, 95% CI 0%–7.1%) for PI‐RADS 3, 9/241 (3.7%, 95% CI 1.7%–7.0%) for PI‐RADS 4 and 1/188 (0.5%, 95% CI 0.0%–2.9%) for PI‐RADS 5 (Figure 3A). Of note, the single PI‐RADS 5 case had an anterior lesion with ISUP 1 disease on targeted biopsy, and ISUP 2 on anterior template biopsies on the same side as the lesion.

**FIGURE 3:**
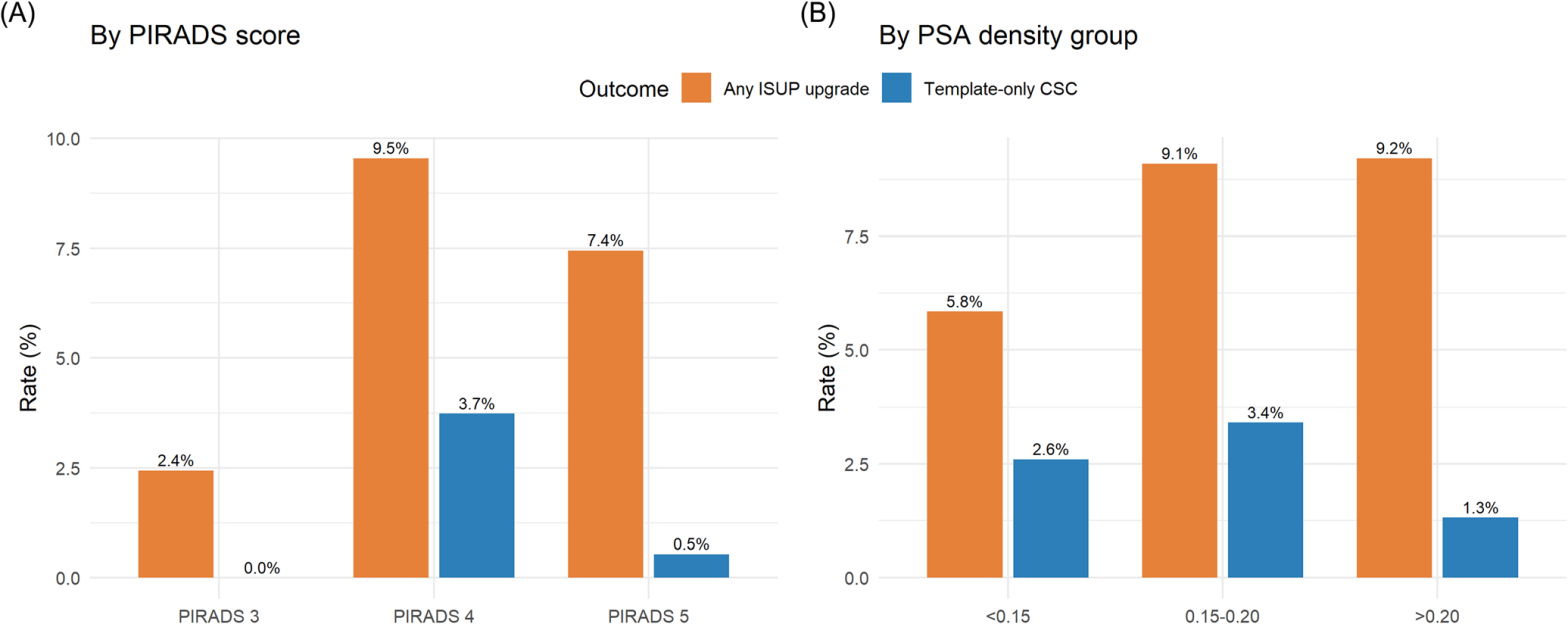
Template only csPCa and any‐upgrade rates by (A) highest PI‐RADS category and (B) PSA density group.

By PSAD group, rates were 4/154 (2.6%, 95% CI 0.7%–6.5%) for PSAD < 0.15, 3/88 (3.4%, 95% CI 0.7%–9.6%) for PSAD 0.15–0.20 and 3/228 (1.3%, 95% CI 0.3%–3.8%) for PSAD > 0.20 (Figure 3B).

### 3.5 Combined PI‐RADS × PSAD subgroups and candidate low‐risk combinations

We cross‐tabulated PI‐RADS category with PSAD group (Figure 4) to attempt to identify sub‐groups with zero observed primary‐outcome events. No patient with a PI‐RADS 3 lesion on MRI was upgraded to csPCa on template biopsy; however the small sample size (*n* = 41) means this should be interpreted with caution.

**FIGURE 4:**
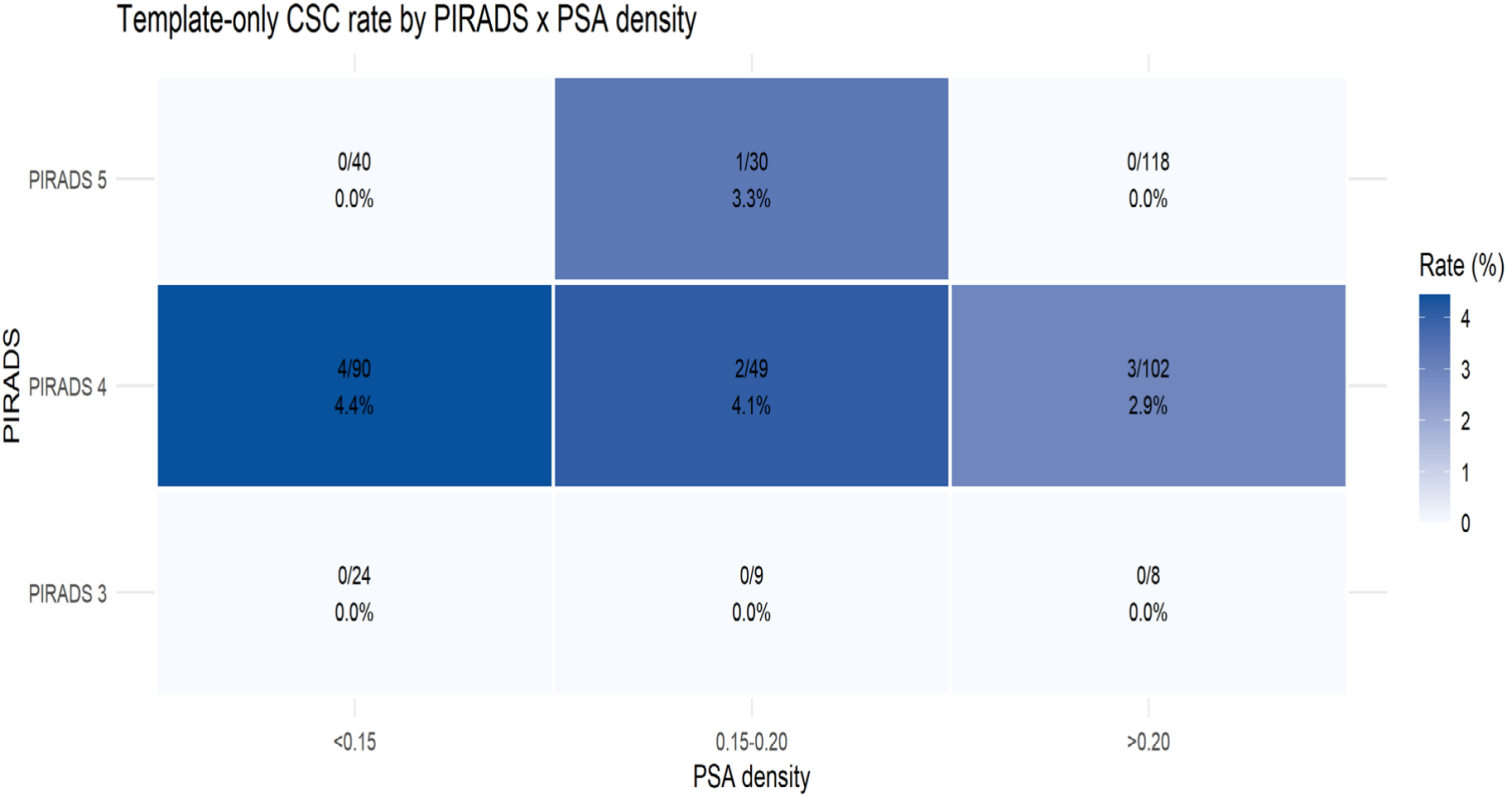
Cross‐tabulation of PI‐RADS score and PSA density.

The largest incremental value of template biopsy was seen in the PI‐RADS 4 group, with csPCa found on template biopsy only in 3.7% of patients, with patients in each PSAD group. Only one patient with a PI‐RADS 5 lesion was upgraded to csPCa by template biopsy alone in the 0.15–0.2 PSAD group.

The largest subgroup in our cohort was patients with a PI‐RADS 5 lesion with PSAD > 0.20 (*n* = 118); there was no incremental value of template biopsy in this subgroup. In this cohort, it was specifically the combination of the most suspicious MRI appearance *together with* the most elevated PSAD—rather than a low PSAD—that was associated with negligible incremental yield from template biopsy.

### 3.6 Incremental value by index lesion size

The primary outcome rate also varied by index lesion size: 6/99 (6.1%, 95% CI 2.3%–12.7%) for lesions < 10 mm, 3/245 (1.2%, 95% CI 0.3%–3.5%) for 10–20 mm and 1/86 (1.2%, 95% CI 0.0%–6.3%) for > 20 mm (Table 3). The highest template‐only detection rate occurred in PI‐RADS 4 lesions smaller than 10 mm (6/93, 6.5%), consistent with the univariable regression finding that smaller index lesion size is the only statistically significant predictor of a missed csPCa on targeted biopsy.

**TABLE 3:** Template‐only csPCa detection rate by index lesion size.

Lesion size	*N*	Events (template‐only csPCa)	Rate (%)	95% CI (%)
< 10 mm	99	6	6.1%	2.3%–12.7%
10–20 mm	245	3	1.2%	0.3%–3.5%
> 20 mm	86	1	1.2%	0.0%–6.3%

*Note*: csPCa = clinically significant prostate cancer (ISUP ≥ 2). CI: exact (Clopper–Pearson) 95% confidence interval.

No template‐only csPCa was detected in PI‐RADS 5 lesions ≤ 20 mm in size (0/97 for 10–20 mm); the single PI‐RADS 5 event occurred in the > 20 mm subgroup (1/86, 1.2%).

### 3.7 Predictors of clinically significant cancer missed by targeted biopsy

On univariate regression, smaller index lesion size was the only predictor significantly associated with the primary outcome (OR 0.50 per 5 mm increase, 95% CI 0.28–0.90, *p* = 0.021); that is, men with smaller lesions were approximately twice as likely to have csPCa missed by targeted biopsy. Prostate volume showed a non‐significant trend toward higher risk with larger glands (OR 1.19 per 10 mL, 95% CI 0.99–1.43, *p* = 0.067). PI‐RADS category, PSA, PSAD and age were not significantly associated with the primary outcome in univariable analysis.

Multivariable modelling of the primary outcome was not undertaken because only 10 primary‐outcome events were observed, which was insufficient for a stable model and risked overfitting. An exploratory multivariable logistic regression model was therefore fitted for the secondary outcome of any ISUP upgrade on template biopsy (38 events), adjusting for PI‐RADS category, PSAD, age and prostate volume (Table 4). No predictor reached statistical significance; this model should be regarded as hypothesis‐generating.

**TABLE 4:** Exploratory multivariable logistic regression—outcome: any ISUP upgrade on template biopsy (38 events).

Characteristic	OR	95% CI	*p*‐value
PI‐RADS category			
PI‐RADS 3	— (reference)	—	
PI‐RADS 4	3.92	0.77–71.6	0.2
PI‐RADS 5	2.68	0.49–50.1	0.4
PSA density (per unit increase)	1.04	0.23–2.13	> 0.9
Age (per year)	1.03	0.98–1.09	0.2
Prostate volume (per mL)	1.00	0.99–1.02	0.7

*Note*: All predictors non‐significant; model is exploratory and should be regarded as hypothesis‐generating. PI‐RADS 3 is the reference category.

Abbreviations: CI = confidence interval; OR = odds ratio.

## 4 DISCUSSION

In this cohort of 470 men undergoing combined targeted and template TPPB, systematic template biopsy detected csPCa that targeted biopsy alone would have missed in 2.1% of all men, representing 2.8% of all csPCa diagnoses made in the cohort. This sits toward the lower end of the wide range reported in the literature of 1.1%–11%.^4^, ^5^, ^6^, ^11^ Our figures are similar to the 1.1–1.5% ‘added value’ reported for contralateral systematic sampling in large transrectal fusion‐biopsy cohorts.^4^, ^12^ Some cohorts, such as a UK series reporting csPCa missed by targeted biopsy in 9%–11% of men, included men with at least one prior negative or low‐grade biopsy (85/104 men, 82%),^5^ a population in whom residual disease outside the previously sampled area is more likely. Given our cohort only included biopsy‐naïve men, it is logical that the yield of template biopsy is lower than this, which aligns with a 2022 study which included both biopsy‐naïve men and men with a prior negative biopsy.^13^

Although the incremental yield was modest, the breakdown of ‘any ISUP upgrade’ on template biopsy (8.1% of men) is clinically informative. Just over half of all upgrades (20/38, 52.6%) occurred in men already known to have csPCa from targeted biopsy, where template biopsy revealed an even higher grade. This does not change the diagnosis of csPCa but may still influence risk stratification, staging imaging decision‐making and treatment selection (for example, determining suitability for active surveillance, focal therapy or radical treatment). The laterality findings add nuance to the ongoing debate about omitting contralateral systematic sampling.^7^, ^12^, ^14^ Within the small primary‐outcome group (*n* = 10), the additional disease was equally likely to be ipsilateral or contralateral to the index lesion, in contrast to several larger series reporting contralateral‐exclusive csPCa in only 1%–2% of men and a clear ipsilateral predominance among template‐detected disease.^4^, ^12^ However, when we examined the larger group of any‐upgrade cases (*n* = 38), ipsilateral predominance was clear and statistically significant (73.7% ipsilateral, *p* = 0.005). This discrepancy most likely reflects the small number of strict primary‐outcome events rather than a true difference and underscores why some clinicians now favour retaining ipsilateral or perilesional biopsies.^7^, ^12^, ^14^

Our most important finding was in our largest subgroup, where there were no additional cases of csPCa found in patients with a PI‐RADS 5 lesion and a PSAD > 0.20 (*n* = 118, exact one‐sided 95% upper bound 2.5%). Only 1 patient (0.5%) in our cohort with a PI‐RADS 5 lesion was upgraded to csPCa on systematic biopsy; this patient had a PSAD of 0.19. This rate is much lower than that described by Sheweita et al.^15^ (35%). This is likely a reflection of our exceptionally high rate of csPCa detection with targeted biopsy (91.5% vs. 76%), despite similar age, PSA and PSAD across the two groups. We obtained slightly more target cores than our UK counterparts (6 vs. 4), however this difference is unlikely to be explained by this alone. Our finding also contrasts with Malewski et al., who identified a PSAD threshold as low as 0.17 ng/mL^2^ as predictive of csPCa detected on systematic biopsy in PI‐RADS 5 lesions^6^; the reason for this discordance is unclear, but may again reflect the exceptionally high targeted‐biopsy detection rate in our PI‐RADS 5 subgroup. A recent transperineal series by Wang et al. also identified PSAD ≥ 0.20 as a threshold associated with reduced incremental yield of systematic biopsy^16^; however, their cohort included PI‐RADS 3–5 lesions and still showed a residual grade group ≥ 2 yield of 7.2% at this threshold, appreciably higher than the near‐zero yield we observed when PSAD ≥ 0.20 was combined with a PI‐RADS 5 lesion specifically. This raises the possibility that PI‐RADS category and PSAD need to be considered jointly, rather than PSAD alone, when selecting candidates for omission of systematic biopsy.

The 2024 EAU guidelines recommend that targeted biopsy should be combined with perilesional sampling, and it should “permit omission of contralateral systematic biopsy” in men with a single PI‐RADS ≥ 4 lesion, or a PI‐RADS 3 lesion with high clinical suspicion of csPCa.^7^, ^14^ The patient groups who will most likely benefit from added systematic biopsies have not yet been clearly identified. The same UK multicentre cohort described above (Sheweita et al., 2611 biopsy‐naïve men undergoing combined targeted and systematic transperineal biopsy) suggested a targeted biopsy‐only approach should be considered for all PI‐RADS 5 lesions and PI‐RADS 4 lesions in the presence of a PSAD greater than 0.15.^15^

A recent multicentre analysis of 2852 men across three tertiary centres found that targeted biopsy combined with perilesional sampling achieved csPCa detection rates equivalent to targeted plus full systematic biopsy in PI‐RADS 4 and PI‐RADS 5 lesions (53% vs. 50%, and 76% vs. 75%, respectively).^17^

Among men with PI‐RADS 4 lesions, by contrast, our rate of csPCa detection was 67.6%, which increased to 71.4% with a combined strategy. Systematic biopsy detected an additional 3.7% of csPCa cases, and there was no significant trend seen across PSAD subgroups. We believe this 3.7% yield in patients with PI‐RADS 4 lesions still warrants further sampling outside of just the target lesion; however our data is not powered to demonstrate whether perilesional or full systematic sampling is warranted in our population.

The rate of csPCa seen in our cohort, both overall (75.7%) and within individual PI‐RADS subgroups, is higher than that reported in much of the literature. The overall rate is partially explained by the nature of our selective, biopsy‐naïve cohort—including only high‐risk lesions and with relatively few PI‐RADS 3 lesions (in whom systematic biopsies only were often performed).

Among patients with a PI‐RADS 4 lesion, our csPCa detection rate (71.4%) exceeds rates seen in other cohorts. The cohort selection may also play a role here, but variation in radiographic and pathological assessment locally should also be considered. In a large cohort of patients undergoing MRI‐TRUS fusion biopsy, prostate cancer and csPCa detection varied significantly between individual radiologists.^18^ Additionally, in a multicentre analysis the positive predictive value of a given PI‐RADS score varied widely across centres^19^—likely as a result of variation in radiological or pathological reporting, or both.

The only statistically significant predictor of csPCa missed by targeted biopsy was a smaller PI‐RADS lesion size (OR 0.50, 95% CI 0.28–0.9). This is biologically plausible: it is more difficult to sample smaller lesions precisely and may, in some cases, represent only the MRI‐visible component of a larger or more heterogeneous tumour of which the extent may be better captured by broader template sampling. This finding is consistent with reports identifying smaller lesion size and lower PI‐RADS as predictors of ‘systematic‐biopsy zero‐benefit’ status among men already diagnosed by targeted biopsy.^20^ Drawing definitive conclusions from this is difficult given the small sample size of events (10 patients with csPCa missed by target biopsy); however we believe lesion size should be another variable considered by clinicians when deciding whether to perform a systematic biopsy.

One final question not addressed by this study, but worth considering, is what is the true oncologic risk to these patients who have a higher ISUP grade disease on systematic biopsy? A recent study of 6588 patients found that when patients had discordant target and systematic biopsy results, the oncological risk (determined by advanced stage, adverse pathology and biochemical recurrence) fell *between* the two biopsy results—rather than following the higher of the two.^21^ The implication of this for our cohort is that the true oncological impact for patients with a higher biopsy grade on systematic biopsy may be less than the raw numbers of our data suggest.

There are pragmatic reasons to minimise systematic sampling where it is safe to do so. Reducing the number of systematic biopsies may offer several benefits for patients, clinicians and for the environment. These benefits include shorter procedure times, fewer specimen pots and less consumable waste, a reduced pathology workload and less detection of clinically insignificant prostate cancer.^15^, ^22^

### 4.1 Clinical implications

In summary, these findings do not support complete abandonment of template biopsy in the MRI‐targeted era, consistent with current EAU and AUA guidance to combine targeted with perilesional or systematic sampling.^7^ They do, however, support a more selective approach. Specifically, our data suggest that template biopsy could reasonably be considered for omission, or replaced with limited perilesional sampling, in men with a PI‐RADS 5 lesion and a PSAD above 0.20 ng/mL^2^, a combination representing one in four men in this cohort (118/470, 25.1%) and associated with no missed csPCa. Template biopsy retains clearer value in men with a non‐significant or negative targeted biopsy result and in men with smaller index lesions. A strategy that risk‐stratifies the *intensity* of systematic sampling—for example, retaining full template biopsy in target‐negative men and those with small or PI‐RADS 4 lesions while reducing it to ipsilateral or perilesional sampling alone in men with large, high‐density PI‐RADS 5 disease—may offer a reasonable compromise between diagnostic completeness and procedural burden (core number, procedure time and associated discomfort), consistent with the direction already taken in recent EAU guidance toward perilesional rather than full systematic sampling.^7^, ^14^

### 4.2 Strengths and limitations

This study has several strengths. All men underwent both targeted and template biopsy in the same session with no selective omission of either component, avoiding a common source of selection bias in the existing literature. The biopsies were performed by only two consultant urologists and utilising the same technique, providing a consistent biopsy approach.

This study also has important limitations. It is a retrospective, single‐centre analysis, and its ethnic composition and biopsy pathway and technique may not generalise to other settings.

As stated above, the selection of only biopsy‐naïve patients undergoing targeted *and* systematic biopsy resulted in under‐representation of patients with PIRADS 3 lesions (due to systematic only approaches in many cases). This selection strategy, in addition to potential variations in radiological, urologist, and pathological technique and reporting, may contribute to the high rates of csPCa seen in our cohort.

Most importantly, the primary outcome was rare (10 events), which constrains the precision of our stratified estimates, which means these analyses should be regarded as hypothesis‐generating rather than practice‐defining. Finally, our outcome was defined at the level of biopsy histology rather than longer‐term oncological endpoints (recurrence, metastasis or survival), so we cannot directly quantify the clinical consequence of the small number of cancers that would have been missed under a targeted‐only strategy.

## 5 CONCLUSIONS

In this cohort of men undergoing combined MRI‐targeted and template TPPB, systematic template biopsy provided an incremental diagnostic yield for csPCa of 2.1% overall, increasing to 8.1% for any ISUP upgrade. Smaller PI‐RADS lesion size was the only independent predictor of csPCa missed by MRI‐targeted biopsy.

Template biopsy provided no incremental diagnostic value among men with PI‐RADS 5 lesions and a PSAD > 0.20 ng/mL^2^. Validation in larger, multicentre cohorts is warranted before these findings can be incorporated into future guideline recommendations.

## AUTHOR CONTRIBUTIONS

**Josh Tiro**: Conceptualisation; data curation; methodology; writing – original draft; writing – review and editing. **Christian Kirk**: Data curation; writing – original draft; writing – review and editing. **Finley Breeze**: Formal analysis. **Amir Zarrabi**: Writing – review and editing. **Luiz Gustavo Modelli de Andrade**: Data curation; formal analysis. **Paulo Roberto Kawano**: data curation; formal analysis. **Flavio Vasconcelos Ordones**: writing – review and editing; supervision. **Lodewikus Vermeulen**: conceptualisation; methodology; writing – original draft; writing – review and editing; supervision.

## CONFLICT OF INTEREST STATEMENT

The authors declare no conflicts of interest.

## Data Availability

De‐identified data may be made available by the corresponding author upon reasonable request and subject to institutional approval.
